# Assessment of risk factors in dogs with presumptive advanced canine cognitive dysfunction

**DOI:** 10.3389/fvets.2022.958488

**Published:** 2022-10-18

**Authors:** Brittany MacQuiddy, Julie A. Moreno, Breonna Kusick, Stephanie McGrath

**Affiliations:** ^1^Department of Clinical Sciences, Colorado State University, Fort Collins, CO, United States; ^2^Department of Environmental and Radiological Health Sciences, Colorado State University, Fort Collins, CO, United States

**Keywords:** canine cognitive dysfunction, neurodegenerative disease, brain, dementia, aging dog

## Abstract

**Objectives:**

The aim of this study was to investigate the potential risk factors involved in the development of presumptive advanced canine cognitive dysfunction (pACCD).

**Materials and methods:**

A questionnaire was developed to identify dogs with presumptive canine cognitive dysfunction (CCD) based on an adapted Canine Dementia Scale and to evaluate for potential risk factors among the presumptive advanced cognitive dysfunction group. The questionnaire was distributed to 7,574 owners of dogs (≥8 years of age) who presented to the CSU VTH between 2017 and 2020. Dogs were classified into four groups based on the Canine Dementia Scale score (normal, mild, moderate, and severe cognitive impairment) and two subgroups for the cognitively impaired groups based on the presence or absence of underlying medical conditions. Comparisons between normal and presumptive advanced cognitively impaired groups, with and without underlying medical conditions, were made against various risk factors. Chi-square tests and logistic regression analysis were used to determine associations between categorical variables and a *p*-value of <0.05 was considered indicative of evidence of association.

**Results:**

The completed response rate for the questionnaire was 14.2% (1,079/7,574). Among those, 231 dogs were classified as having presumptive advanced cognitive dysfunction. The prevalence of presumptive advanced cognitive dysfunction in the included age groups was 8.1% in ages 8 to <11 years, 18.8% in ages 11 to <13 years, 45.3% in ages 13 to <15 years, 67.3% in ages 15 to <17 years, and 80% in ages >17 years. Dogs with a thin body condition score had the largest contribution to the chi-square statistic. Based on the logistic regression model, both age (*p* < 0.001) and BCS (*p* = 0.0057) are associated with presumptive ACCD.

**Conclusion and relevance:**

The chi-square test and logistic regression analysis both suggested an association between a thin body condition and an increased chance of cognitive decline. However, it is difficult to determine if the thin BCS in this group could be secondary to another confounding factor. The prevalence of cognitive dysfunction rapidly increased with age in this study. These findings warrant continued studies including veterinary evaluations to explore risk factors of canine dementia.

## Introduction

Canine cognitive dysfunction syndrome (CCD) is a progressive neurodegenerative disease seen in the aging dog. Dogs with CCD have been suggested as a feasible spontaneous surrogate for Alzheimer's disease (AD) in humans, as they share many similarities in the neuropathological changes associated with the disease process (1–3). Additionally, dogs are exposed to the same environment as their human counterparts, making an ideal model for research into the environmental risk factors for neurodegenerative disease. Due to the increasing longevity of canines and humans, neurodegenerative diseases are becoming more prevalent. In 2016, there were ~77 million owned dogs in America with 15% (just over 11.5 million) of the population ≥11 years of age (4). The estimated prevalence of CCD ranges from 14% to over 60%, increasing as the dog ages (5). One study describes that the prevalence of CCD in dogs 11–12 years of age was 28% and in dogs 15–16 years of age was 68% (6–10). Behavior changes attributed to CCD in dogs can be represented by the acronym DISHAA: disorientation, alterations in interactions, changes in sleep-wake cycle, house soiling, alterations in activity levels, and anxiety level changes (11). Many of these behavior changes go unreported by owners as they are thought to be part of the normal aging process; therefore, client education and thorough historical investigation are important. In a survey of nearly 500 dogs aged 8–19 years of age, the overall prevalence of CCD was 14.2% with only 1.9% of the cases having been previously diagnosed by a veterinarian (6, 7).

Common pathological brain abnormalities noted in both humans and dogs with cognitive dysfunction include cerebrovascular disease, amyloid beta (Aβ) accumulation, oxidative brain damage, neuronal mitochondrial dysfunction, glutamate-mediated excitotoxic neuronal damage, impaired neuronal glucose metabolism, microglial activation, and astrocyte dysfunction (1, 5, 12–14). The two classical features most discussed in humans with AD are Aβ deposition and the accumulation of hyperphosphorylated tau leading to neurofibrillary tangles (NFTs). Dogs with cognitive impairment have been shown to have increased Aβ accumulation in the brain with decreased plasma and cerebrospinal fluid Aβ compared with age-matched healthy dogs (1–3, 5, 14–16). In previous studies, NFTs were either completely absent in the cohort of CCD dogs or present in <10% of the study population (1, 3, 5, 17, 18). More recent efforts have shown success in identifying early evidence of tau protein accumulation in CCD dogs with similar regional distributions as in humans with dementia (1). It is postulated that dogs may not live long enough to develop NFTs or a different antibody other than AT8 (standard marker in humans) is required to identify them (1, 5). Ongoing investigation of the neurodegenerative process, including neuropathological changes and signaling pathways, and the clinical progression of the disease is needed to make an early and accurate diagnosis, identify potential targets for treatments, and apply preventive measures.

Currently, diagnosis of CCD is an antemortem diagnosis of exclusion. Medical conditions that can cause behavioral changes include the following: endocrine disorders, sensory dysfunction (blindness/deafness), metabolic disorders, pain, gastrointestinal disease, urogenital conditions, dermatologic disease, or intracranial abnormalities (i.e., brain neoplasms) (6). All potential causes of behavior changes must be ruled out prior to diagnosing CCD. An initial evaluation may include physical/neurologic/orthopedic examinations, bloodwork (complete blood count, chemistry, thyroid panel), blood pressure, thoracic radiographs, and abdominal ultrasound. If these diagnostics are normal then next diagnostic steps would include a brain MRI to evaluate for structural changes and a cerebrospinal fluid analysis to evaluate for evidence of inflammation, infection, or neoplasia. In addition to these diagnostics, there are screening tools available for CCD that have been previously validated. The most widely used screening tools are the Canine Cognitive Dysfunction Rating scale (CCDR) and the CAnine DEmentia Scale (CADES). The CADES has been shown to be a better assay to separate different stages of CCD, identify earlier stages of cognitive impairment, and follow progression of disease compared with the CCDR scale (19). More recent studies evaluating the use of CADES questionnaire combined with plasma biomarkers, such as neurofilament light chain or Aβ concentrations, may more readily predict CCD in elderly dogs (15, 20, 21). The use of one of these scale ratings as a screening tool and the previously mentioned diagnostics to rule out underlying medical conditions are currently the best methods to evaluate for CCD antemortem.

Risk factors described in humans with AD include the following: increasing age, genetics, head injury, environmental (air pollution, diet, heavy metals, infections), obesity, diabetes mellitus, and cardiovascular disease (22). Some risk factors have been explored in dogs with CCD including age, sex, breed, breed size, reproductive status, nutrition, exercise, housing, and air pollution. Across studies the only common finding is that increasing age significantly increases the risk for developing CCD. One study found that dogs fed a controlled high-quality diet were 2.8 times less likely to develop CCD when compared with dogs fed an uncontrolled low quality diet (23). In one study evaluating cognitive dysfunction in dogs with and without idiopathic epilepsy, there was a higher risk of developing CCD at a younger age in the idiopathic epileptic subset of dogs (24). Another study evaluating sex, reproductive status, breed size, and vitamin E levels found no variable to be statistically significant between normal and cognitively impaired groups of dogs (25). More studies with larger sample sizes are needed to further elucidate all the potential risk factors for CCD in canine patients. This knowledge could have future implications for lifestyle changes or preventive measures that could be implemented to slow progression or even prevent cognitive impairment in the future.

## Materials and methods

### Questionnaire

A questionnaire consisting of 32 questions was created using Qualtrics^1^ software (see Supplementary material). The purpose of the questionnaire was to identify dogs with presumptive CCD and to evaluate for potential risk factors in the presumptive advanced CCD groups. The Canine Dementia Scale (CADES) proposed by Madari et al. was used in this study, but with minor wording changes to improve clarity for owners (19). The adapted CADES questionnaire can be found in Supplementary materials. The CADES tool is composed of 17 items scored from 0 to 5 (depending on the frequency of the behavior) to evaluate spatial orientation, social interactions, sleep-wake cycles, and house soiling. Permission was obtained for the use of the CADES screening tool in this study. Other questions evaluated for the age, sex, breed, reproductive status, weight as it relates to breed size, body condition score (BCS) as assessed by the owner, energy level, underlying medical conditions, diet, environment (community- rural vs. suburban vs. urban vs. large urban areas, number of dogs in the household, smoking household), previous head trauma, and the length of time with the current owner. These questions were used to assess for potential risk factors involved in the development of presumptive advanced CCD in this population of dogs. Once created, the entirety of the questionnaire including the CADES portion was evaluated by a small focus group at Colorado State University (veterinarians, veterinary technicians, and doctors of philosophy) that provided feedback regarding phrasing of the questions which was incorporated in the final version.

### Participants

Participants for the online survey were identified by performing a medical record search of dogs (≥8 years of age) that had presented to the CSU VTH from October 2017 to October 2020. An email was sent using Qualtrics software to 7,574 dog owners requesting their participation in our survey. The survey was made available between November 23, 2020 and January 27, 2021. One thousand seventy-nine anonymous surveys were completed and included in the descriptive analysis. Approval from the CSU Institutional Review Board was obtained to distribute the survey (IRB protocol #2076). All owners were presented with a statement of informed consent prior to taking the survey.

### Data recording and analysis

Data were reviewed from completed surveys (1,079). The data were exported from Qualtrics to a Microsoft Excel^2^ spreadsheet. Responses were divided into four groups: 1. Normal/not affected (NA) group (CADES score <8); 2. Mild cognitive impairment (MiCI) group (CADES score 8–23); 3. Moderate cognitive impairment (MoCI) group (CADES score 24–44); 4. Severe cognitive impairment (SCI) group (CADES score >44). Groups 2–4 were further divided into subgroup A (+MC) dogs *with* underlying medical conditions or subgroup B dogs *without* underlying medical conditions. This was considered necessary as this was an online-survey without access to full medical history or records, making it possible that underlying medical conditions reported by owners were a cause for the behavior changes noted in this cohort of dogs.

The prevalence of presumptive CCD was calculated in all dogs both including and excluding those with underlying medical conditions to incorporate a prevalence range. This was done to account for the lack of in-person evaluations as the true prevalence likely falls within this range. Prevalence of presumptive advanced CCD was also assessed across different age groups. Exact ages of the population were not collected. Instead, they were categorized into age ranges. Initially, there were five ranges as part of the questionnaire: 8 to <11 years, 11 to <13 years, 13 to <15 years, 15 to <17 years, and >17 years of age. These ranges were used when calculating and presenting prevalence of presumptive CCD. When evaluating for risk factors across different ages, all dogs were classified into three groups based on estimated life expectancies: 8 to <11 years (short-lived), 11 to <13 years (medium-lived), and ≥13 years of age (long-lived) (7, 26).

The group definitions and abbreviations can be seen in Table 1. Descriptive comparisons were made between all groups (NA, MiCI+/-MC, MoCI+/-MC, and SCI+/-MC). Chi-square was used to determine associations between two groups (NA and pACCD) and different assessed risk factors such as sex/reproductive status (female spayed vs. female intact vs. male castrated vs. male intact), breed size (small ≤35 lbs vs. medium = 36–55 lbs vs. large ≥56 lbs), body condition score (thin vs. average vs. overweight), energy level (high >90 min/day vs. moderate = 45–90 min/day vs. low <45 min/day of outdoor activity), diet (commercial vs. non-commercial = homemade/raw food), community (rural = population <2,500 vs. suburban = population of 2,500 to <500,000 vs. urban = population of 500,000 to <1,000,000 vs. large urban = population >1,000,000), number of dogs in the household (1 vs. 2+), smoking household (yes vs no), and previous head trauma (yes vs. no vs. I don't know-unknown history) as independent variables. A *p*-value of <0.05 was considered significant. The MoCI+/-MC and SCI+/-MC groups were combined to form the pACCD group. This was done for ease of interpreting chi square results and is further explained in the discussion section. Additionally, chi-square was also used to determine associations of potential risk factors and the NA and pACCD group without underlying medical conditions.

**Table 1:** Definition of each group and the number of dogs per group out of the number of completed surveys.

**Groups**	**# of dogs per group/completed surveys**	**% of dogs in this group**
Group 1: Normal/not affected (NA)	543/1,079	50.3%
Group 2A: Mild cognitive impairment w/ underlying medical conditions (MiCI+MC)	271/1,079	25.1%
Group 2B: Mild cognitive impairment w/out underlying medical conditions (MiCI)	34/1,079	3.2%
Group 3A: Moderate cognitive impairment w/ underlying medical conditions (MoCI+MC)	151/1,079	14%
Group 3B: Moderate cognitive impairment w/out underlying medical conditions (MoCI)	14/1,079	1.3%
Group 4A: Severe cognitive impairment w/ underlying medical conditions (SCI+MC)	52/1,079	4.8%
Group 4B: Severe cognitive impairment w/out underlying medical conditions (SCI)	14/1,079	1.3%

Multivariate logistic regression analysis was used to assess relationships between CCD status (NA or pACCD) as the response and the statistically significant risk factors (determined by the chi-square test) as the predictors including age (see Supplementary material). This was to determine an association when accounting for age. Dogs with underlying medical conditions were included in these analyses. The risk of cognitive dysfunction was assessed to be positive if the estimate odds ratio was >1. A *p*-value of <0.05 was considered evidence of association.

## Results

The total response rate was 23.7% (1,798/7,574). Seven hundred nineteen questionnaires were incomplete and excluded from analysis. The completed response rate for the questionnaire was 14.2% (1,079/7,574). Of the completed surveys, 543 dogs had CADES scores <8 and were classified into group 1 (NA). The remaining dogs were divided into groups according to the degree of cognitive impairment and whether there were underlying medical conditions reported (Table 1).

**Figure 1:**
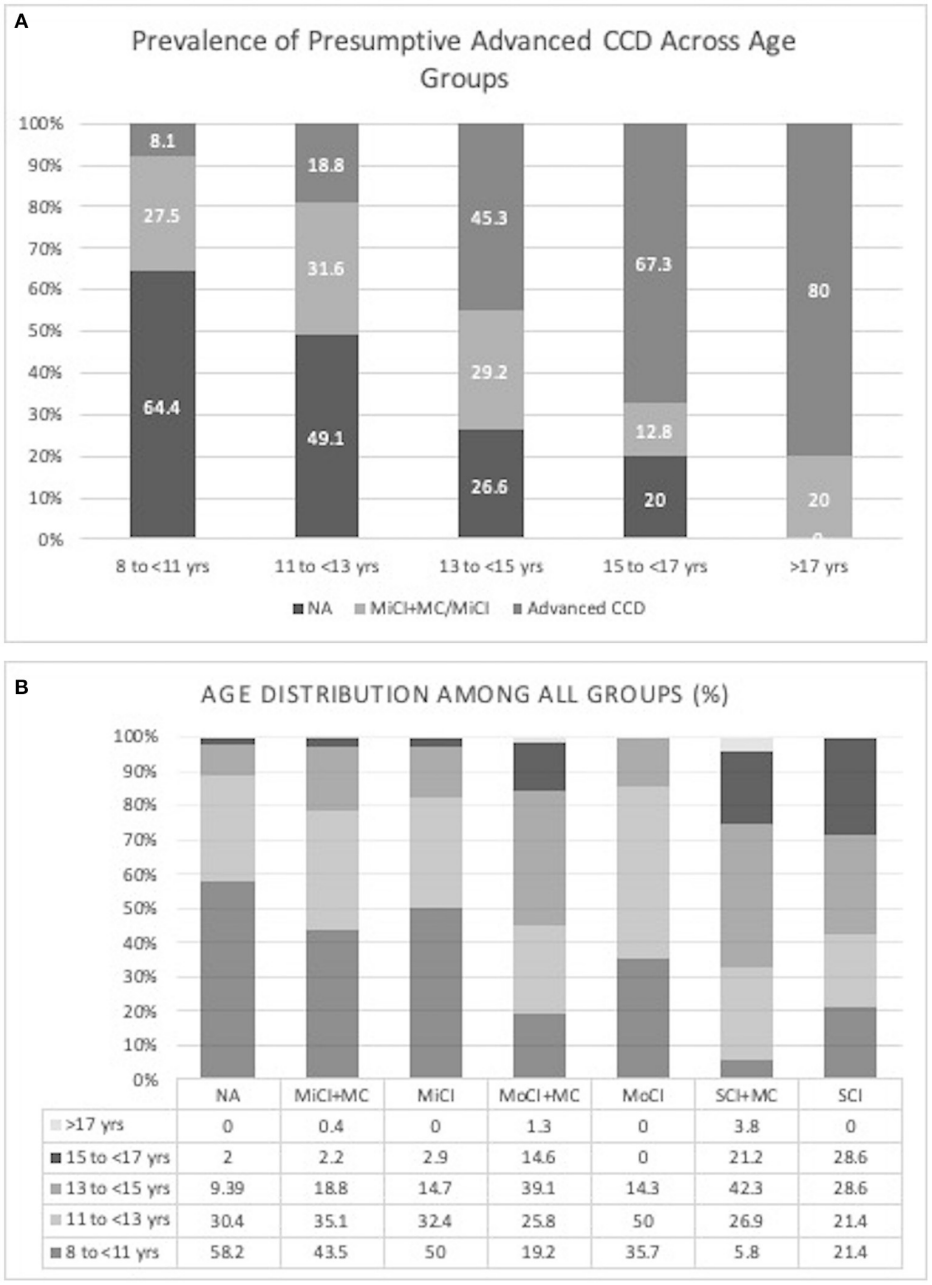
**(A)** Prevalence of presumptive CCD across age groups (NA, Normal/Not affected; MiCI + MC, mild cognitive impairment with underlying medical conditions; MiCI, mild cognitive impairment without underlying medical conditions; Advanced CCD, moderate and severe cognitive impairment with and without underlying medical conditions), **(B)** Age distribution among all groups (%) (NA, Normal/Not affected; MiCI + MC, mild cognitive impairment with underlying medical conditions; MiCI, mild cognitive impairment without underlying medical conditions; MoCI + MC, moderate cognitive impairment with underlying medical conditions; MoCI, moderate cognitive impairment without underlying medical conditions; SCI + MC, severe cognitive impairment with underlying medical conditions; SCI, severe cognitive impairment without underlying medical conditions).

The prevalence of all stages of presumptive CCD in this cohort of dogs without underlying medical conditions was 5.7% which increased to 49.7% when including dogs with comorbidities, based on their CADES score alone. The prevalence of pACCD in different age groups was 8.1% in ages 8 to <11 years, 18.8% in ages 11 to <13 years, 45.3% in ages 13 to <15 years, 67.3% in ages 15 to <17 years, and 80% in ages >17 years (Figure 1A). The age distribution among the different groups can be seen in Figure 1B. When asked if their dog had ever been diagnosed with CCD before, 1.8% said yes (19/1,079) with 94.7% of them having pACCD. There were also 2.2% who reported they weren't sure if their dog had ever been diagnosed with CCD before. Among this group, 62.5% had evidence of pACCD based on their CADES score.

When asked how long they had owned their pet, only 1.3% (14/1,079) of all owners had owned the dog for <6 months. The majority of households had at least two or more dogs at home; however, there was no evidence of association of dogs living in a multi-dog household and the development of presumptive advanced CCD (chi-square *p*-value = 0.97). The sex and reproductive status of all dogs in the study was 50.3% spayed females, 0.8% intact females, 46.2% castrated males, and 2.7% intact males. Among the intact female population, 37.5% had evidence of pACCD (Table 2). When considering the sex or reproductive status (male vs. female; intact vs. neutered) vs. two groups (NA and pACCD), the chi-square *p*-values were 0.48 and 0.43, respectively (df = 2 for both).

**Table 2:** The distribution of risk factors across all study groups.

**Distribution across all groups (%)**		**#dogs/total study population (%)**	**NA**	**MiCI + MC**	**MiCI**	**MoCI + MC**	**MoCI**	**SCI + MC**	**SCI**	**pACCD**
Sex/repro	Female spayed	50.3	50.4	23.4	3.7	14.7	1.1	5.0	1.7	22.5
	Female intact	0.8	50.0	12.5	0.0	12.5	0.0	25.0	0.0	37.5
	Male castrated	46.2	49.3	27.9	2.4	13.8	1.6	4.0	1.0	20.4
	Male intact	2.7	65.5	13.8	6.9	3.5	0.0	10.3	0.0	13.8
BCS	Average	74.7	53.1	24.7	3.6	12.1	1.4	4.0	1.1	18.6
	Overweight	12.2	44.7	31.1	0.7	16.7	0.7	6.1	0.0	23.5
	Thin	13.1	39.7	22.0	2.8	22.0	1.4	8.5	3.6	35.5
Breed size	Small (≤35 lbs)	37.0	43.9	25.0	4.0	16.3	1.50	7.8	1.5	27.1
	Medium (36–55 lbs)	25.4	51.8	23.7	3.3	15.0	1.1	3.6	1.5	21.2
	Large (≥56 lbs)	37.6	55.7	26.1	2.2	11.1	1.2	2.7	1	16
Energy level	High	5.7	75.4	18.0	3.3	3.3	0.0	0.0	0.0	3.3
	Moderate	45.4	62.8	23.7	3.5	7.0	1.0	2.0	0.0	10.0
	Low	48.9	35.8	27.3	2.8	21.8	1.7	8.0	2.6	34.1
#dogs in the household	1	36.5	48.7	26.9	3.6	15.7	0.8	3.3	1.0	20.8
	2+	63.5	51.2	24.1	2.9	13.0	1.6	5.7	1.5	21.8
Community	Rural	18.3	55.8	25.0	2.5	11.2	1.0	2.5	2.0	16.7
	Suburban	66.5	49.3	24.8	3.0	15.2	1.3	5.4	1.0	22.9
	Urban	7.4	47.4	28.8	4.9	8.8	1.3	6.3	2.5	18.9
	Large urban	7.8	48.8	24.9	3.6	15.5	2.4	3.6	1.2	22.7
Smoking household	Yes	2.6	39.3	35.7	0.0	17.8	3.6	3.6	0.0	25.0
	No	97.4	50.6	24.8	3.2	14.0	1.2	4.9	1.3	21.4
Previous head trauma	Yes	2.5	37.0	37.0	0.0	14.9	3.7	7.4	0.0	26.0
	No	86.3	51.8	24.7	3.3	13.4	1.2	4.5	1.1	20.2
	Unknown	11.2	42.1	25.6	2.5	18.2	1.7	6.6	3.3	29.8
Diet	Commercial	92.8	50.5	24.9	3.2	13.9	1.5	4.7	1.3	21.4
	Non-commercial	7.2	47.4	28.2	1.3	15.4	0.0	6.4	1.3	23.1

There were 138 breeds represented in this study overall. The most common breeds included mixed breed (190/1,079), Kuvasz (83/1,079), Golden Retriever (48/1,079), Border Collie (47/1,079), Australian Shepherd (37/1,079), Chihuahua (34/1,079), German Shepherd (33/1,079), and Labrador Retriever (31/1,079). The breed with the highest incidence of pACCD was the Chihuahua at 35.3% (12/34). The weights reported were used to categorize the population into small (≤35 lbs), medium (36–55 lbs), or large (≥56 lbs) breed dogs. When considering the breed size vs. two groups (NA and pACCD), the chi-square *p*-value = 0.0001792 (df = 2). The body conditions scores as assessed by the owner were 74.7% average (806/1,079), 12.2% overweight (132/1,079), and 13.1% thin (141/1,079). Approximately 35.5% of the thin BCS group showed pACCD compared with 18.6% in the average BCS group and 23.5% in the overweight BCS group (Table 2). Considering BCS (thin, average, overweight) vs. NA and pACCD, the chi-square *p*-value = 0.00003929 (df = 2).

The reported energy levels were 5.7% high (61/1,079), 45.4% moderate (490/1,079), and 48.9% low (528/1,079). The highest incidence of pACCD was 34.1 % in the low energy group followed by 10% in the moderate and 3.3% in the high energy group. There was no association between the dogs' energy level vs. the NA and pACCD groups (chi-square *p*-value = 0.31). The majority of dogs (66.5%; 718/1,079) lived in a suburban area with no evidence of differences in incidence of CCD in dogs from rural, urban, or large urban communities (chi-square *p*-value = 0.14).

Only 2.5% of dogs (27/1,079) were reported to have a known history of head trauma with no evidence of higher incidence of CCD in this group. There was no association regarding the dogs with previous head trauma vs. the NA and pACCD groups (chi-square *p*-value = 0.24). Approximately 92.8% of owners said they feed their dog either a commercial only diet or a mixture of commercial food with or without homemade cooked/raw food. There was no evidence of higher incidence of CCD in the small percentage of dogs that were fed a non-commercial food diet (chi-square *p*-value = 0.63). Twenty-eight dogs (2.6%) were reported to come from a smoking household with 25% having pACCD compared with 21.4% of dogs in non-smoking households (Table 2). There was no evidence of association regarding dogs that live in a smoking household vs. the NA and pACCD groups (chi-square *p*-value = 0.40). The variables of living in a smoking household, previous head trauma, and diet lack statistical power due to the disproportionate numbers among the different categories.

The highest scoring CADES category in the presumptive mild and moderate CI groups was sleep-wake cycle compared with spatial orientation in the presumptive severe CI groups (Table 3).

**Table 3:** Mean scores for each CADES category among all presumptive CI groups.

**Mean scores**	**Mild w underlying MC**	**Mild + no underlying MC**	**Mod w underlying MC**	**Mod + no underlying MC**	**Severe w underlying MC**	**Severe + no underlying MC**
Spatial orientation	1.82	1.38	6.66	8.29	15.02	18.43
Sleep-wake cycle	5.36	6.18	11.09	10	11.21	18.43
Social interaction	4.42	3.56	8.58	8.64	12.63	15.14
House soiling	2.41	1.88	6.5	6.71	12.38	14

Both breed size and BCS were independently determined to have evidence of an association between the NA and pACCD groups. Small breed dogs had the largest contribution to the chi-square statistic. Dogs with a thin BCS (NA and pACCD) had the largest contribution to the chi-square statistic. The number of observed small breed dogs with pACCD compared with the expected value were 108 and 84.5, respectively (Table 4). The number of observed pACCD dogs with a thin BCS compared with the expected value were 50 and 31.6, respectively (Table 5).

**Table 4:** Evaluation of the association of breed size between NA and presumptive Advanced CCD groups using the chi-square test [degrees of freedom: 2; chi-square (χ^2^): 17.2539; *p*-value: 0.0001792].

		**Small (≤ 35 lbs)**	**Medium (36–55 lbs)**	**Large (≥56 lbs)**	**Total observed**
NA	Observed	175	142	226	543
	Expected	198.539	140.31	204.151	
	χ^2^ contribution	2.79076	0.0203538	2.33833	
Presumptive advanced CCD	Observed	108	58	65	231
	Expected	84.4612	59.6899	86.8488	
	χ^2^ contribution	6.56009	0.0478446	5.49658	
Total observed		283	200	291	774

**Table 5:** Evaluation of the association of body condition score between NA and presumptive Advanced CCD groups using the chi-square test [degrees of freedom: 2; chi-square (χ^2^): 20.2895; *p*-value: 0.00003928].

		**Thin**	**Average**	**Overweight**	**Total observed**
NA	Observed	56	428	59	543
	Expected	74.3643	405.496	63.1395	
	χ^2^ contribution	4.53509	1.2489	0.271395	
Presumptive advanced CCD	Observed	50	150	31	231
	Expected	31.6357	172.504	26.8605	
	χ^2^ contribution	10.6604	2.93573	0.637954	
Total observed		283	200	291	774

When calculating the chi-square statistic for the proposed risk factors in the pACCD group without underlying medical conditions compared to the NA group, the only association found was BCS (chi-square *p*-value = 0.0344; df = 2). Again, dogs with a thin BCS (NA and pACCD without underlying medical conditions) had the largest contribution to the chi-square statistic. Breed size was not determined to have an association when removing those with underlying conditions (Table 6).

**Table 6:** Evaluation of the association of body condition score between NA and presumptive Advanced CCD groups without underlying medical conditions using the chi-square test [degrees of freedom: 2; chi-square (χ^2^): 6.7394 *p*-value: 0.0344].

		**Thin**	**Average**	**Overweight**	**Total observed**
NA	Observed	56	428	59	543
	Expected	59.91	426.03	57.06	
	χ^2^ contribution	0.26	0.01	0.07	
Presumptive advanced CCD w/out underlying medical conditions	Observed	7	20	1	28
	Expected	3.09	21.97	2.94	
	χ^2^ contribution	4.95	0.18	1.28	
Total observed		63	448	60	571

A logistic regression model was performed with CCD status (NA or pACCD) as the response and including both age (8 to <11 years vs. 11 to <13 years vs. ≥13 years of age) and BCS (thin vs. average vs. overweight) as predictors. Based on the logistic regression model, both age (*p*-value < 0.001) and BCS (*p*-value = 0.0057) are associated with presumptive ACCD. Specifically, the estimated odds of presumptive ACCD are higher for those with a thin BCS compared to those with an average BCS (OR = 2.212, *p*-value = 0.0048).

Similarly, a logistic regression model was performed with CCD status (NA or pACCD) as the response and including both age (as described above) and breed size (small ≤35 lbs vs. medium = 36–55 lbs vs. large ≥56 lbs) as predictors. Based on this model, age is associated with CCD status (*p*-value < 0.001) but breed size is not associated with CCD status (*p*-value = 0.5268). When assessing the distribution of breed size across the different ages in the NA and pACCD groups, the majority of the population ≥13 years of age were small breed dogs (53.7%; 102/190). Two-thirds of this population had presumptive advanced CCD.

## Discussion

Canine cognitive dysfunction syndrome is a neurodegenerative disorder of aging dogs in which potential risk factors are still poorly understood. Canine cognitive dysfunction and AD in humans are multifactorial diseases with both internal and external risk factors (5, 17, 23, 27, 28). Studies previously performed to explore risk factors in dogs vary widely and are largely contradictory to each other. For example, one study found that there was no association between sex and size of the breed to the development of CCD (25), while another study stated that females and small sized dogs seemed to be at a higher risk for CCD (8). Since the purpose of this study was to identify potential risk factors associated with presumptive CCD, the dogs with moderate (MoCI ± MC) and severe (SCI ± MC) cognitive impairment were classified as the presumptive advanced CCD group which were used in the statistical evaluation of possible risk factors. Mild cognitive impairment in humans is considered the intermediate stage between cognitive decline associated with normal aging and more advanced decline of cognitive function, which was also stated in the development of the CADES scale (25, 29).

Canine cognitive dysfunction is a commonly underdiagnosed syndrome, as mild signs of cognitive impairment are often unreported. In this study, 1.8% of the presumptive cognitively impaired dogs reported a previous diagnosis of CCD, which is consistent with previous findings (7). In this study as well as earlier studies, the prevalence of CCD has shown to increase with age (5–10). The prevalence of pACCD in this study may be overestimated, as dogs with underlying medical conditions were not excluded. Further work is needed to classify underlying medical conditions and their contribution to behavior changes seen in elderly dogs. This would allow future studies to differentiate between dogs with underlying medical conditions that do or do not have CCD. The true overall prevalence of presumptive CCD among the current dog population may, in fact, lie somewhere between the low estimate of 5.7% and the high estimate of 49.7%, as there is likely a portion of the dogs with underlying medical conditions that have concurrent CCD. While the current estimations of prevalence of presumptive CCD were made with the use of a previously validated screening tool (CADES), in-person evaluations with targeted diagnostics would be needed to confirm these findings.

Regarding sex and reproductive status, it was found that the prevalence of pACCD was the highest among intact females with females overall having a higher prevalence than males. However, there was no evidence of association regarding this risk factor. In the Azkona et al. study, females and neutered dogs were shown to have a higher prevalence of CCD than male dogs and intact dogs, respectively (8). The majority of the dogs in the current study were neutered with only 3.5% representing intact dogs. The uneven distribution in dogs surveyed could have potentially influenced the results. A large-scale study involving an even distribution of dogs across sex and reproductive status would be needed for understanding the true risk among these groups.

There were 138 breeds represented across all groups in this study with mixed breed being the most commonly reported followed by the Kuvasz. According to a 2020 report by the American Kennel Club, the Labrador Retriever was the number one most commonly owned dog with Kuvasz coming in at number 177 (30). The breed list used in this study was obtained from the AKC website and represented as a drop-down selection in the online survey (31). Kuvasz answer choice was directly above that of the Labrador Retriever. It is suspected that a technical error was made and most dogs reported to be Kuvasz were meant to be reported as Labrador Retrievers; however, due to the nature of the study, we could not confirm this suspicion. This could be easily overcome with phone or in-person evaluations.

A large-scale study assessing for a correlation between specific dog breeds and prevalence of CCD has yet to be performed. A previous study of 957 dogs found no correlation between prevalence of CCD and breed size but did not comment on specific breeds (7). The breed of each dog was recorded in this study but, based on the potential error when selecting a breed in the questionnaire, it is difficult to make any conclusions. Additionally, due to the variability in size of dogs based on breed, a relationship between weight and CCD has yet to be identified (32). In one study evaluating 1.3 million people worldwide, it was found that a higher body mass index at mid-life was correlated with a higher risk of developing dementia later in life (33). In previous reports where small breed dogs were assessed to have a higher rate of CCD, it was suspected that this was largely attributed to the fact that small breed dogs have a longer lifespan than large breed dogs; therefore, they live long enough to develop CCD (23, 34). However, a more recent study, evaluating over 4,000 dogs with 66 breeds represented, found that all breeds had a similar cognitive course regardless of breed size or related lifespan. In other words, larger dogs seemed to have a limited decline in cognition at the end of their comparatively shorter lifespan. A limitation of the study by Watowich et al. was that they only had a very small portion of dogs >11 years of age (35). In the current study, breed size was assessed in three categories (≤35, 36–55, and ≥56 lbs). This was roughly based on classifications of small, medium, and large breed dogs by the AKC with no overlapping categories (36). Based on a chi-square *p*-value = 0.001792, there was evidence of association between breed size when comparing the NA and pACCD groups. However, breed size was not found to be associated when performing the chi-square test in pACCD group without underlying medical conditions or when using the logistic regression model. Over 53% of the dogs ≥13 years of age were small breeds which likely skewed the results of the chi-square test that showed association. In the future, a larger focus should be placed on correlating specific breeds to both breed size and the BCS of the patient based on veterinary assessment.

When evaluating BCS as a risk factor, 74.7% of owners reported that their dog had an average body condition. In a strictly online-survey, caution must be taken when interpreting this result as it is common for clients to underestimate their pet's BCS. Keeping that in mind, there were more pACCD dogs (with and without underlying medical conditions) with a thin BCS than expected based on the chi-square tests. This suggests that there is an association between thin BCS and an increased chance of cognitive dysfunction which was also found in the logistic regression model. As previously stated, a higher body mass index in people at mid-life has been correlated with a higher risk of cognitive decline in people later in life (33). The current study only evaluated the BCS of the dogs at the time of the survey when, based on the human data, it may have been more beneficial to ask about the dogs BCS at middle age. The middle age of the dog would have to consider the lifespan of the animal which is variable among small and large breeds. It is difficult to determine based on the data alone if there were underlying disease processes contributing to the thin BCS in this group of dogs which could be contributing to the behavioral changes or if there was also underlying cognitive dysfunction. To the author's knowledge, no other studies have shown a potential relationship between a thin BCS and CCD. Future studies assessing dogs at middle age, taking into consideration the lifespan of the animal, and then again as seniors would be needed to determine if the findings in people also apply to the canine population.

The most commonly reported energy level in this cohort of dogs was a low energy level (49%). This was defined as <45 min of outdoor activity per day. This was also the group with the highest incidence of presumptive CCD at 34.1% which was 3.4 times higher than the moderate energy group and over 10 times higher than the high energy group. However, there was no evidence of association when evaluated energy level as a risk factor. It has been postulated that engaging in activities requiring physical exercise throughout life, or even at early stages of cognitive dysfunction, to stimulate the cognitive pathways may promote neuroprotection and prevent age-related cognitive decline (37). This is one of the potential risk factors in which intervention from the owner could slow or even prevent onset of cognitive dysfunction. There was no evidence from this data to suggest that diet, single vs. multi-dog household, community, smoking vs. non-smoking household, or evidence of previous head trauma had any correlation with the onset of presumptive CCD. However, in the case of the diet, specifications for the type of commercial food fed such as if it was appropriate for the age and breed of the dog were not made. Therefore, based on the current data and lack of statistical power, diet/nutrition cannot be excluded as a potential risk factor for the development of CCD. In one study that did account for a well-balanced controlled diet vs. an uncontrolled diet, they found that dogs on a controlled diet were 2.8 times less likely to develop cognitive dysfunction (23).

Across the four domains evaluated by the CADES, the domain with the highest mean score in the presumptive mild/moderate CI groups was sleep-wake cycle and in the presumptive severe CI groups was spatial orientation. In one study, the most commonly affected domains were social interactions and sleep-wake cycle in aged dogs (19). However, a variety of different phenotypic manifestations can be identified indicating that CCD is a disease with multi-domain clinical impairment (19, 38).

Limitations of this study are largely attributed to the method used to conduct the survey. More specific limitations regarding the reliability of the data and potential risk factors are mentioned throughout the discussion. As this was a voluntary online-survey, the time it takes to complete the survey was kept between 5 and 10 min to maximize the response rate; however, this limited the amount of data that could be analyzed. For those that did respond, a large number only answered the first few questions which did not include any questions regarding behavior, environment or medical history. Therefore, these incomplete surveys were excluded from analysis. The survey was emailed to owners of dogs ≥8 years of age that had presented to the CSU VTH within the previous 3 years. While best efforts were made so as not to alert the owner specifically about CCD, there were a small portion of owners that did not complete the CADES portion of the survey. One possible reason for this could be that the owners did not believe their dog had behavior changes at home; therefore, they may have thought it unnecessary to complete this portion. It is also possible that they did not understand the questions in the CADES portion which is why they were left blank. The inability to follow up with owners about their incomplete responses makes it difficult to understand other variables involved. Estimates of prevalence of pACCD were made based on the completed surveys but were likely overestimated, as dogs with underlying medical conditions were not completely eliminated from analysis. It is important to highlight that this population of dogs did show neurobehavioral changes, as reported by their owners, that could be consistent with CCD; however, only a presumptive diagnosis was made due to the absence of in-person evaluations. Therefore, potential risk factors identified in this study remain unclear and require further evaluation. In-person appointments to complete, at minimum, physical examinations, including neurologic and orthopedic examinations, and to review veterinary medical records would have substantially increased the power of this study.

## Conclusions

The survey was completed by 1,079 dog owners and identified 536 dogs ≥8 years of age that had a CADES score of at least 8 or more, which indicated evidence of presumptive cognitive impairment. The highest scoring CADES category in the presumptive mild and moderate CI groups was sleep-wake cycle compared with spatial orientation in the presumptive severe CI groups. The prevalence of pACCD in the included age groups was 8.1% in ages 8 to <11 years, 18.8% in ages 11 to <13 years, 45.3% in ages 13 to <15 years, 67.3% in ages 15 to <17 years, and 80% in ages >17 years. The high occurrence of presumptive CCD in dogs over 8 years of age, and even more prominent in dogs over 11 years, confirms the importance of further research in this field. Based on the chi-square tests including dogs with underlying medical conditions, thin BCS and small breed dogs were suggested to be independently associated with an increased chance of developing CCD. However, based on the chi-square tests that included only those dogs without underlying medical conditions, thin BCS was the only risk factor that showed an association with CCD. Thin BCS was also shown to have an association with CCD in the logistic regression analysis with age as a predictor. Due to the limitations of the study, only a presumptive diagnosis of CCD could be made in this population of dogs; therefore, the validity of the results remains unclear. The contribution to a thin BCS by a confounding factor cannot be ruled out. Additional studies that include veterinary evaluations are needed to further elucidate the risk factors associated with cognitive decline in the aging canine population.

## Data availability statement

The original contributions presented in the study are included in the article/Supplementary material, further inquiries can be directed to the corresponding author.

## Ethics statement

The animal study was reviewed and approved by Colorado State University's Institutional Review Board. Written informed consent was obtained from the owners for the participation of their animals in this study.

## Author contributions

BM, JM, BK, and SM all contributed to developing the questionnaire. BM analyzed and interpreted the data in addition to writing the manuscript. SM and JM participated in manuscript edits/feedback. All authors read and approved the final manuscript.

## Conflict of interest

The authors declare that the research was conducted in the absence of any commercial or financial relationships that could be construed as a potential conflict of interest.

## Publisher's note

All claims expressed in this article are solely those of the authors and do not necessarily represent those of their affiliated organizations, or those of the publisher, the editors and the reviewers. Any product that may be evaluated in this article, or claim that may be made by its manufacturer, is not guaranteed or endorsed by the publisher.

## Footnotes

^1^Version January 2021 of Qualtrics. Copyright © 2021 Qualtrics. Qualtrics and all other Qualtrics product or service names are registered trademarks or trademarks of Qualtrics, Provo, UT, USA. https://www.qualtrics.com.

^2^Version 15.33 (170409) of Microsoft Excel. Copyright © 2017 Microsoft. All rights reserved. Redmond, WA, USA.
