# Supplementary material for: Assessment of risk factors in dogs with presumptive advanced canine cognitive dysfunction
Source: Front Vet Sci. 2022 Oct 18;9:958488. doi: 10.3389/fvets.2022.958488 (PMC9622924; doi:10.3389/fvets.2022.958488)
Supplement: Supplementary file 2 [file Table_2.DOCX]

CCD Logistic Regression Analysis

Ann Hess

2022-09-02

InData <- read_excel("C:/hess/Consulting-2022/McGrath/CCD.9.1.22.xlsx",
 sheet = "Sheet3")
str(InData)

## tibble [774 × 4] (S3: tbl_df/tbl/data.frame)
## $ Age : num [1:774] 11 11 11 11 11 13 11 8 11 8 ...
## $ BCS : num [1:774] 2 1 2 1 2 2 2 2 2 2 ...
## $ Size: chr [1:774] "3" "2" "1" "1" ...
## $ CCD : num [1:774] 0 0 0 0 0 0 0 0 0 0 ...

InData$Age <- as.factor(InData$Age)
InData$BCS <- as.factor(InData$BCS)
InData$Size <- as.factor(InData$Size)

## Summary Tables:

table(InData$CCD, InData$Age)

##
## 8 11 13
## 0 316 165 62
## 1 40 63 128

table(InData$CCD, InData$BCS)

##
## 1 2 3
## 0 56 428 59
## 1 50 150 31

table(InData$CCD, InData$Size)

##
## 1 2 3
## 0 175 142 226
## 1 108 58 65

table(InData$Size, InData$Age)

##
## 8 11 13
## 1 102 79 102
## 2 93 56 51
## 3 161 93 37

## CCD vs BCS accounting for Age

BCSmodel <- glm(CCD ~ BCS + Age, family = binomial, data = InData)
Anova(BCSmodel)

## Analysis of Deviance Table (Type II tests)
##
## Response: CCD
## LR Chisq Df Pr(>Chisq)
## BCS 10.53 2 0.005171 **
## Age 175.93 2 < 2.2e-16 ***
## ---
## Signif. codes: 0 '***' 0.001 '**' 0.01 '*' 0.05 '.' 0.1 ' ' 1

emmeans(BCSmodel, pairwise ~ BCS, type = "response")

## $emmeans
## BCS prob SE df asymp.LCL asymp.UCL
## 1 0.464 0.0569 Inf 0.356 0.576
## 2 0.282 0.0219 Inf 0.241 0.326
## 3 0.367 0.0599 Inf 0.259 0.490
##
## Results are averaged over the levels of: Age
## Confidence level used: 0.95
## Intervals are back-transformed from the logit scale
##
## $contrasts
## contrast odds.ratio SE df null z.ratio p.value
## 1 / 2 2.212 0.560 Inf 1 3.136 0.0049
## 1 / 3 1.494 0.515 Inf 1 1.164 0.4746
## 2 / 3 0.675 0.189 Inf 1 -1.402 0.3396
##
## Results are averaged over the levels of: Age
## P value adjustment: tukey method for comparing a family of 3 estimates
## Tests are performed on the log odds ratio scale

## CCD vs Size accounting for Age

Sizemodel <- glm(CCD ~ Size + Age, family = binomial, data = InData)
Anova(Sizemodel)

## Analysis of Deviance Table (Type II tests)
##
## Response: CCD
## LR Chisq Df Pr(>Chisq)
## Size 1.282 2 0.5268
## Age 168.592 2 <2e-16 ***
## ---
## Signif. codes: 0 '***' 0.001 '**' 0.01 '*' 0.05 '.' 0.1 ' ' 1

emmeans(Sizemodel, pairwise ~ Size, type = "response")

## $emmeans
## Size prob SE df asymp.LCL asymp.UCL
## 1 0.345 0.0325 Inf 0.285 0.411
## 2 0.302 0.0377 Inf 0.233 0.380
## 3 0.297 0.0328 Inf 0.237 0.365
##
## Results are averaged over the levels of: Age
## Confidence level used: 0.95
## Intervals are back-transformed from the logit scale
##
## $contrasts
## contrast odds.ratio SE df null z.ratio p.value
## 1 / 2 1.22 0.279 Inf 1 0.870 0.6594
## 1 / 3 1.25 0.269 Inf 1 1.030 0.5576
## 2 / 3 1.02 0.244 Inf 1 0.097 0.9949
##
## Results are averaged over the levels of: Age
## P value adjustment: tukey method for comparing a family of 3 estimates
## Tests are performed on the log odds ratio scale
