# Supplementary material for: Assessment of risk factors in dogs with presumptive advanced canine cognitive dysfunction
Source: Front Vet Sci. 2022 Oct 18;9:958488. doi: 10.3389/fvets.2022.958488 (PMC9622924; doi:10.3389/fvets.2022.958488)
Supplement: Supplementary file 3 [file Table_3.DOCX]

Canine Cognitive Dysfunction Survey

**Consent to Participate**
I understand that this research is being conducted by students and faculty from Colorado State University (College of Veterinary Medicine & Biomedical Sciences). The goal of this survey is to understand the signs associated with canine aging. We invite you to complete this short, anonymous online survey. Your participation in this research is voluntary. When data is shared with the public, it will be in combined data pools and no individual information will be shared. There are no direct benefits or known risks associated with participation in this survey. We anticipate this survey will take no longer than 10 minutes to complete. If you have any questions about the research, please contact our team at VTH_seniorpetstudies@colostate.edu. If you have questions about your rights as a volunteer, contact the CSU IRB atRICRO_IRB@mail.colostate.edu or 970.491.1553.

Q1 Do you agree to the terms of the Consent to Participate?

- I agree
- I disagree, I understand that by disagreeing to the disclosure agreement I cannot participate in this study

Q2 Is your dog living or deceased?

- Living
- Deceased

Q3 How many dogs are in your household?

- 1
- 2
- 3
- 4
- 5
- 6
- >6

Q4 How old is your dog?

- <1 year
- 1 to <3 years
- 3 to <5 years
- 5 to <8 years
- 8 to <11 years
- 11 to <13 years
- 13 to <15 years
- 15 to <17 years
- >17 years

Q5 How long have you owned your dog?

- <6 months
- 6 months to <1 year
- 1 to <3 years
- 3 to <5 years
- 5 to <8 years
- 8 to <10 years
- >10 years

**Demographic Information**

Q6 What is the sex and neuter status of your dog?

- Female intact (unaltered)
- Female spayed (fixed)
- Male intact (unaltered)
- Male castrated (fixed)

Q7 What is your dog's weight in pounds?

- <10 pounds
- 10-35 pounds
- 36-55 pounds
- 56-75 pounds
- >75 pounds

Q8 Which of the following body conditions best describes your dog?

- Thin
- Average
- Overweight

Q9 What is your dogs level of activity?

- Low energy (<45 minutes of outdoor activity per day)
- Moderate energy (45- 90 minutes of outdoor activity per day)
- High energy (>90 minutes of outdoor activity per day)

Q10 What breed is your dog?

Choose the most representative breed

▼ Mixed Breed (1) ... Yorkshire Terrier (277)

Q11 Does your dog have any of the following underlying/ongoing medical conditions? (Check all that apply)

- Digestive system issues (dental disease, megaesophagus, gastroesophageal reflux, food sensitivities, inflammatory bowel disease, pica, chronic pancreatitis, chronic diarrhea or vomiting)
- Endocrinopathies (hypothyroidism, diabetes, cushing's disease, addison's disease, insulinoma, etc)
- Urinary disease (urinary tract infections, urinary crystals/stones, kidney disease, etc)
- Heart or lung disease (dilated cardiomyopathy, arrhythmia, pulmonary hypertension, recurrent pneumonia, chronic bronchitis, etc)
- Musculoskeletal disease (arthritis, previously broken bone that didn't heal appropriately, congenital malformation, or any disease that affects mobility)
- Neurologic disease (seizures, brain tumor, stroke, balance issues (vestibular disease), congenital malformations of the brain/spinal cord, spinal cord or disc disease, sleep disorders)
- Sensory dysfunction (vision or hearing impairment/loss)
- Dermatologic disease (skin allergies, chronic ear/skin infections, etc)
- Ongoing behavior issues since puppy or young adult (aggression, separation anxiety, submissive/anxious urination, etc)
- Neoplasia (Cancer- malignant or aggressive forms)
- No underlying conditions/Healthy
- Other ________________________________________________

Q12 Does your dog have any previous history of head trauma?

- Yes
- No
- I don't know

Q13 Has your dog been diagnosed with Cognitive Dysfunction? Examples of this would be Dog Alzheimer’s or Dog Dementia

- Yes
- No
- I'm not sure

Q14 What diet is your dog fed? (Choose all that apply)

- Commercial dog food
- Homemade cooked diet
- Raw diet

Q15 Which of the following best describes the setting where you and your dog live?

- Large Urban areas (large metropolitan area with population >1,000,000)
- Urban area (metropolitan area with population >500,000 but <1,000,000)
- Suburban areas (population >2,500 but less than <500,00)
- Rural areas (population <2,500)

Q16 Does your dog live in a smoking household?

- Yes
- No

Behavior changes in aging dogs

Q17-33 Please choose the frequency at which your dog exhibits the following... (The following should be answered with one dog in mind)

|  | Never | At least once in the last 6 months | At least once per month | 2-4 times per month | Several times a week |
| --- | --- | --- | --- | --- | --- |
| Appears lost or confused in a familiar environment (inside/outside) |  |  |  |  |  |
| Has difficulty recognizing familiar people and/or animals inside or outside the home |  |  |  |  |  |
| Abnormally responds (increased or decreased) to familiar objects |  |  |  |  |  |
| Wanders around without purpose or direction |  |  |  |  |  |
| Has difficulty performing previously learned tasks |  |  |  |  |  |
| Has abnormal night-time behaviors (wandering, vocalization, restlessness) |  |  |  |  |  |
| Unable to sleep at night and/or sleeps most of the day |  |  |  |  |  |
| Changes in interactions with people or other animals (welcoming, petting, playing) |  |  |  |  |  |
| Changes in individual behavior of your dog (exploration behavior, play, performance) |  |  |  |  |  |
| Decreased response to commands |  |  |  |  |  |
| Irritable |  |  |  |  |  |
| Expressions of aggression |  |  |  |  |  |
| Eliminates (urinates/defecates) at home at random locations |  |  |  |  |  |
| Eliminates in his/her kennel or sleeping area |  |  |  |  |  |
| Decreased signaling to go outside to eliminate |  |  |  |  |  |
| Eliminates indoors after a recent walk outside |  |  |  |  |  |
| Eliminates at uncommon outdoor locations (concrete) |  |  |  |  |  |
